# Regenerative Cartilage Treatment for Focal Chondral Defects in the Knee: Focus on Marrow-Stimulating and Cell-Based Scaffold Approaches

**DOI:** 10.3390/cells14151217

**Published:** 2025-08-07

**Authors:** Filippo Migliorini, Francesco Simeone, Tommaso Bardazzi, Michael Kurt Memminger, Gennaro Pipino, Raju Vaishya, Nicola Maffulli

**Affiliations:** 1 Department of Trauma and Reconstructive Surgery, University Hospital of Halle, Martin-Luther University Halle-Wittenberg, 06097 Halle (Saale), Germany; 2 Department of Orthopaedic and Trauma Surgery, Academic Hospital of Bolzano (SABES-ASDAA), via Lorenz Böhler 5, 39100 Bolzano, Italy; francesco.simeone@sabes.it (F.S.); tommaso.bardazzi@sabes.it (T.B.); michael.memminger@sabes.it (M.K.M.); 3 Department of Life Sciences, Health, and Health Professions, Link Campus University, Via del Casale di San Pio V, 00165 Rome, Italy; 4 Department of Orthopaedics, Villa Erbosa, IRCCS Ospedale S. Raffaele, 40119 Milano, Italy; dottgennaropipino@yahoo.it; 5 Department of Orthopaedics and Joint Replacement Surgery, Indraprastha Apollo Hospital, Sarita Vihar, New Delhi 110076, India; raju.vaishya@gmail.com; 6 Department of Trauma and Orthopaedic Surgery, Faculty of Medicine and Psychology, University “La Sapienza” of Rome, Via di Grottarossa 1035, 00189 Roma, Italy; n.maffulli@qmul.ac.uk; 7 School of Pharmacy and Bioengineering, Keele University Faculty of Medicine, Stoke-on-Trent ST4 7QB, UK; 8 Centre for Sports and Exercise Medicine, Queen Mary University of London, Barts and the London School of Medicine and Dentistry, Mile End Hospital, 275 Bancroft Road, London E1 4DG, UK

**Keywords:** chondral, cartilage, surgery, cell-based therapy, regenerative medicine

## Abstract

Focal chondral defects of the knee are a common cause of pain and functional limitation in active individuals and may predispose to early degenerative joint changes. Given the limited regenerative capacity of hyaline cartilage, biologically based surgical strategies have emerged to promote tissue repair and restore joint function. This narrative review critically examines current treatment approaches that rely on autologous cell sources and scaffold-supported regeneration. Particular emphasis is placed on techniques that stimulate endogenous repair or support chondrocyte-based tissue restoration through the use of autologous biomaterial constructs. The influence of lesion morphology, joint biomechanics, and patient-specific variables on treatment selection is discussed in detail, focusing on the differences between tibiofemoral and patellofemoral involvement. Biologically driven approaches have shown promising mid- to long-term outcomes in selected patients, and are increasingly favoured over traditional methods in specific clinical scenarios. However, the literature remains limited by heterogeneity in study design, follow-up duration, and outcome measures. This review aims to provide an evidence-based, morphology-informed framework to support the clinical decision-making process in the management of knee cartilage defects.

## 1. Introduction

Focal chondral defects of the knee are a frequent source of short-term pain and functional limitation, as well as early-onset joint degeneration in the long term [1,2,3,4,5,6,7,8,9,10]. Focal chondral defects of the knee are more common in active individuals, particularly those affected by traumatic or overload-related injuries [1,2,3,4,11,12,13,14]. The limited regenerative capacity of hyaline cartilage, a tissue devoid of vasculature, innervation, and lymphatics, results in a biological environment where spontaneous repair is rare and generally produces fibrocartilage, which lacks the mechanical and biochemical properties required to withstand normal articular loads [15,16,17,18,19,20].

A comprehensive characterisation of lesion morphology is essential to guide treatment selection and prognostic expectations. Cartilage defects should be distinguished according to their origin and pattern: focal lesions are typically post-traumatic, well circumscribed, and isolated; degenerative lesions tend to be diffuse, irregular, and associated with early osteoarthritic changes [21,22,23]. A second dimension involves lesion symmetry: unipolar defects affect a single articular surface, and kissing lesions involve both femoral and tibial or femoral trochlea and patellar surfaces in direct contact [24]. The extent and distribution should be considered, as single lesions may behave differently from multifocal or multipolar patterns that often reflect broader biomechanical or systemic alterations [25]. These classifications are not merely descriptive but also influence the biological potential for repair, the mechanical environment of the lesion, and the suitability and expected durability of different surgical strategies. Over the past two decades, cartilage repair has evolved from simple marrow stimulation techniques to sophisticated cell-based and scaffold-supported approaches [10,26,27,28,29,30]. Microfractures remain a first-line option for small defects; however, concerns regarding fibrocartilage durability, subchondral bone alterations, and long-term decline in clinical outcomes have prompted the search for more reliable alternatives [31,32,33,34,35]. Among these, autologous matrix-induced chondrogenesis (AMIC) has been proposed as a biological and cost-effective one-step procedure that combines marrow stimulation with a collagen membrane to enhance cellular retention and differentiation [11,36,37,38,39,40,41]. Matrix-induced autologous chondrocyte implantation (mACI), including scaffold- and spheroid-based systems, provides a more robust biological approach to regenerate hyaline-like tissue with enhanced integration and load resistance [12,42,43,44,45,46,47,48,49]. Additionally, emerging techniques involving minced cartilage, enzyme-released chondrons, or mesenchymal stem cell-loaded scaffolds have garnered attention for their potential to simplify procedures while maintaining biological efficacy; however, long-term outcomes remain under investigation [10,50,51,52,53]. The clinical decision-making process must also consider patient-specific factors, including age, body mass index, activity level, knee alignment, ligamentous stability, meniscal integrity, and the chronicity of symptoms, all of which affect tissue quality, healing potential, and failure risk. Therefore, the selection of a surgical technique should not rely solely on defect size or location, but should also reflect the interaction between lesion morphology, joint status, and patient profile.

The purpose of this narrative review is to provide a critical and evidence-based synthesis of current treatment strategies for focal chondral defects of the knee, with a specific focus on bone marrow stimulation techniques and autologous scaffold-based cellular therapies. Emphasis is placed on regenerative approaches that aim to restore hyaline-like cartilage through intrinsic autologous biological stimulation or autologous chondrocyte implantation, providing a framework for morphology-informed treatment decisions based on lesion characteristics and patient-specific factors. Clinical outcomes, therapeutic indications, complications, and limitations are discussed in light of the available literature. Rather than proposing fixed indications, this review aims to offer a flexible framework grounded in current evidence, supporting the development of patient-specific, morphology-informed treatment strategies. This review focuses exclusively on biologically driven cartilage repair strategies based on autologous cell sources, including bone marrow stimulation techniques and scaffold-assisted chondrocyte-based therapies. Procedures involving the structural transplantation of osteochondral tissue, such as allograft or mosaicplasty, were deliberately excluded given their distinct biological rationale, graft-associated immunological considerations, and specific indications, which differ from the regenerative objectives of intrinsic or cell-mediated repair. By restricting the analysis to autologous regenerative modalities, this review aims to provide a homogeneous and mechanistically coherent synthesis of current evidence applicable to morphology-driven cartilage repair in clinical practice.

## 2. Methods

All the clinical studies investigating surgical management for chondral defects of the knee were accessed. Only studies published in peer-reviewed journals were considered. Given the author’s language capabilities, English, German, Italian, French, and Spanish articles were eligible. Studies with levels I to IV of evidence, according to the Oxford Centre of Evidence-Based Medicine [54], were considered. The following algorithm was preliminarily established:

- Problem: Chondral defects of the knee.
- Intervention: Surgical management.
- Outcomes: Clinical outcomes, imaging results, or failure.

In July 2025, the following databases were accessed: PubMed, Web of Science, and Scopus. The Medical Subject Headings (MeSHs) used for the database search are reported in Table 1.

## 3. Biological and Biomechanical Considerations in Cartilage Repair

Articular hyaline cartilage is a highly specialised connective tissue that provides a low-friction, wear-resistant surface for load transmission across diarthrodial joints. It is composed of chondrocytes embedded within an extracellular matrix rich in type II collagen, proteoglycans, and water [19,20,55,56,57]. The architecture of cartilage is zonally organised, with superficial, middle, deep, and calcified zones each contributing to its mechanical function. Despite its durability, cartilage lacks blood vessels, lymphatics, and nerves, which severely limits its intrinsic capacity for repair and regeneration [58,59,60]. Consequently, even small full-thickness defects fail to regenerate functional hyaline tissue and instead heal, if at all, with mechanically inferior fibrocartilage. The subchondral bone plays a critical role in both the pathogenesis of cartilage defects and their potential for repair [61,62]. The osteochondral unit functions as a biomechanical and metabolic continuum, where subchondral changes, such as bone marrow oedema, sclerosis, or cysts, can influence cartilage homeostasis and repair responses. Disruption of this interface, whether through trauma or iatrogenic manipulation, alters load distribution, induces inflammatory signalling, and compromises the structural environment required for stable tissue integration. Following cartilage injury, chondrocytes undergo apoptosis or adopt a catabolic phenotype, releasing matrix-degrading enzymes such as MMPs and aggrecanases [9,63]. Simultaneously, the synovial environment becomes enriched with pro-inflammatory cytokines, including IL-1β and TNF-α, which further inhibit anabolic activity and promote matrix breakdown [10,12,64,65]. In the presence of subchondral disruption, marrow elements infiltrate the defect, triggering the initial formation of a fibrin clot, followed by the recruitment of mesenchymal progenitor cells. These cells have the potential to differentiate into chondrogenic lineages; however, in the absence of structural guidance and stable mechanical conditions, the repair tissue often consists of disorganised fibrocartilage with inferior biomechanical resilience [14,66,67]. The mechanical environment exerts a profound influence on cartilage regeneration. Optimal compressive loading promotes chondrogenic differentiation and matrix synthesis, while excessive shear or instability can disrupt cell viability and integration. Therefore, the success of any cartilage repair technique depends not only on biological stimulation but also on biomechanical containment and joint homeostasis [68,69,70]. Larger or uncontained lesions, or those in malaligned or unstable knees, require more advanced strategies, possibly including scaffold-based cellular implants or combined realignment procedures. Moreover, the repair response is influenced by lesion size, chronicity, and the biological microenvironment. Acute focal defects in young patients with preserved joint architecture have a higher probability of successful regeneration. Conversely, chronic or degenerative lesions often exhibit matrix calcification, chondrocyte senescence, and poor integration capacity, requiring more robust interventions. The bone–cartilage interface itself may also undergo pathological remodelling, including tidemark duplication, vascular invasion and subchondral bone plate thickening, all of which contribute to repair failure. The regeneration of articular cartilage is a biologically and mechanically constrained process, where multiple factors, including cellular activity, matrix integrity, inflammatory signalling, subchondral bone status, and mechanical loading, converge to determine the quality of repair. Any therapeutic approach must address these variables in a patient-specific and lesion-specific manner to optimise clinical outcomes.

## 4. Bone Marrow-Stimulating Techniques

**Figure 1:**
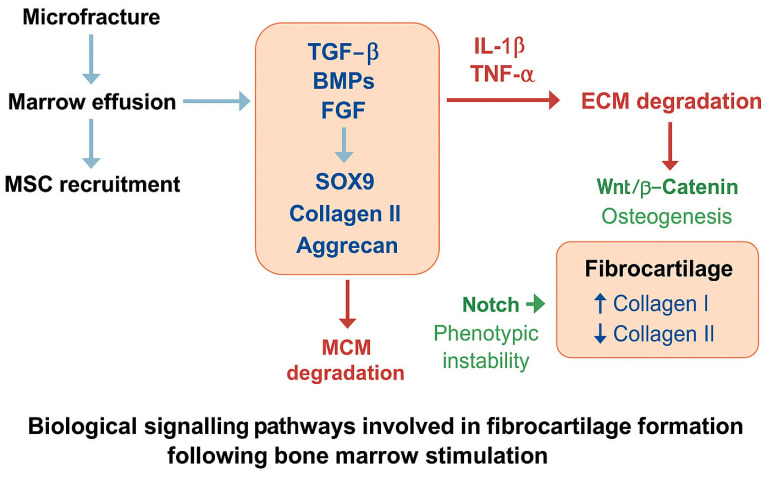
Schematic representation of the key biological signalling pathways involved in fibrocartilage formation following bone marrow stimulation. Microfracture induces marrow effusion and mesenchymal stem cell (MSC) recruitment into the defect site. Chondrogenic differentiation is primarily driven by TGF-β, BMPs, and FGF through SOX9 activation, promoting type II collagen and aggrecan synthesis. However, in the absence of scaffold stability and under inflammatory conditions (IL-1β, TNF-α), matrix-degrading enzymes (MMPs, ADAMTS) are upregulated, leading to extracellular matrix (ECM) breakdown. Wnt/β-catenin and Notch pathways further divert MSCs from stable chondrogenesis, favouring osteogenic transition or phenotypic instability. The resulting repair tissue predominantly consists of fibrocartilage, characterised by increased type I collagen and reduced type II collagen content.

The rationale behind bone marrow stimulation techniques is rooted in the concept of accessing mesenchymal stromal cells from the subchondral bone marrow to initiate a reparative cascade [71,72]. Following subchondral perforation (Figure 1), mesenchymal stem cells (MSCs) migrate from the marrow into the defect, where they encounter a hypoxic and mechanically unstable niche enriched with key morphogens such as transforming growth factor-beta (TGF-β), bone morphogenetic proteins (BMP-2 and BMP-7), and fibroblast growth factor (FGF-2). These signals converge on intracellular pathways, notably the SMAD2/3 axis downstream of TGF-β, which induces the expression of chondrogenic transcription factors and matrix components, including SOX9, COL2A1, and ACAN. Yet in the absence of a stable scaffold and under the influence of pro-inflammatory mediators such as interleukin-1β and tumour necrosis factor-alpha, MSC differentiation often remains incomplete, favouring the deposition of a disorganised fibrocartilage rich in type I collagen and deficient in hyaline-specific markers. Additional regulators, including Wnt/β-catenin and Notch signalling, further skew the repair response, promoting fibrocartilaginous or osteogenic phenotypes when subchondral remodelling or joint instability persist.

**Figure 2:**
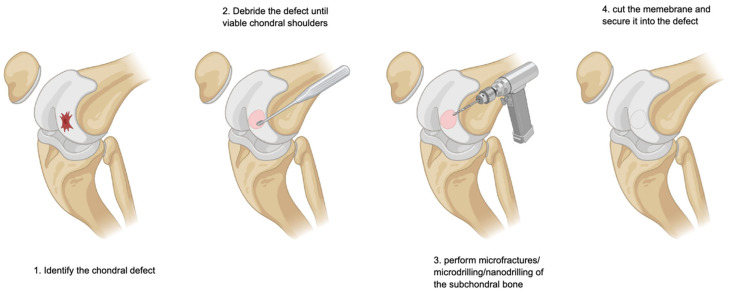
Autologous matrix-induced chondrogenesis.

While the initial response can lead to symptom improvement, the resulting fibrocartilage is mechanically inferior to hyaline cartilage and lacks its durability [35,73]. Microfracture remains the most widely used marrow stimulation procedure, and involves the creation of multiple channels into the subchondral bone using awls or needles [10,74,75,76]. Modifications such as nanofracture or needle-puncture techniques aim to optimise the recruitment of progenitors while reducing collateral damage to the surrounding bone [77]. Nevertheless, these techniques are limited by the instability of the resulting clot and exposure to joint shear forces. To overcome this latter issue, scaffold-augmented bone marrow stimulation was introduced [26,35,78,79,80,81]. The most validated among these is AMIC, which combines microfracture with the application of a collagen type I/III resorbable membrane over the defect (Figure 2) [38,40,42,44]. This scaffold stabilises the clot, enhances progenitor retention, and supports matrix deposition. Preclinical models demonstrate improved integration and tissue quality when using scaffolds. Several studies validated the efficacy of AMIC in focal chondral defects of the knee. A recent meta-analysis of 18 studies (548 patients) found that, at approximately forty months of follow-up, the visual analogue scale (VAS) showed a decrease of −3.9/10, the Tegner Activity Scale showed an increase of +0.8/10, and the Lysholm Knee Scoring System showed an increase of +28.9/100, as did the International Knee Documentation Committee (IKDC) by +33.6/100. At the last follow-up, no patient showed signs of hypertrophy [11]. These values overcome the minimum clinically important difference, attesting their clinical relevance. The rate of failure was 3.8% [11]. To date, we have not been able to identify studies which evaluated AMIC as a revision procedure for chondral defects of the knee. A clinical trial evaluated the clinical outcomes and imaging of 27 AMICs as a revision procedure for failed AMIC surgery for osteochondral defects of the talus [40]. At approximately 4 years, patient-reported outcome measures (PROMs) indicated that patients were moderately satisfied with the procedure; the MOCART score demonstrated a significant improvement from baseline to the last follow-up [40]. Of note, 30% (8 of 27 patients) experienced persistent pain and underwent a further chondral procedure [40]. These results encourage the use of AMIC as a revision procedure for failed AMIC in recurrent symptomatic osteochondral defects [40].

Other techniques include cell-free synthetic scaffolds or hyaluronan-based membranes combined with microfracture, as well as one-step procedures using bone marrow aspirate concentrate (BMAC) [82,83,84,85,86]. However, these remain investigational and lack robust comparative data. In the patellofemoral joint, the application of marrow stimulation techniques poses specific challenges because of the high shear forces and complex biomechanics [87,88]. Microfracture alone has shown poor durability in this region. AMIC, when performed in conjunction with axis correction and patellar tracking optimisation, has shown encouraging results [48,89,90]. Studies suggest its effectiveness in focal defects up to 2.5–3 cm^2^ in the absence of bipolar lesions or severe instability. The scaffold provides crucial mechanical protection in this high-stress compartment. Adjunctive procedures such as tibial tubercle osteotomy or lateral release may be required to optimise outcomes. While results are promising, failure rates remain higher than those observed in lesions of the femoral condyles, underscoring the importance of careful patient selection and surgical planning.

## 5. Scaffold- and Cell-Based Techniques

The development of scaffold- and cell-based cartilage restoration techniques marked a fundamental shift in the management of focal chondral defects of the knee, particularly in lesions exceeding 2 cm^2^ or in patients with failed marrow stimulation procedures [26,91,92]. The biological rationale underlying these approaches stems from the limitations of microfracture and related bone marrow stimulation techniques, which predominantly yield fibrocartilaginous repair tissue. While initially satisfactory, this tissue often lacks long-term durability and integration under physiological loads. Scaffold- and cell-based therapies aim to replicate a hyaline-like architecture and enhance the biomechanical resilience of regenerated cartilage by supporting cellular proliferation, differentiation, and matrix synthesis within a controlled three-dimensional environment [26,84,93,94,95,96]. This strategy aims to bridge the gap between biological efficacy and mechanical competence, providing a tailored approach that considers defect size, location, patient age, and previous interventions [97,98].

**Figure 3:**
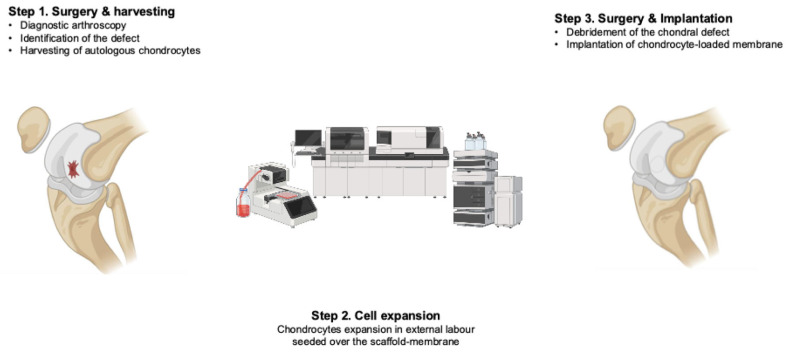
Matrix-induced autologous chondrocyte implantation.

Autologous chondrocyte implantation (ACI) represents the most commonly performed surgical technique for managing chondral defects of the knee [39,99,100,101,102,103]. In ACI procedures, an arthroscopy of the knee is performed to assess cartilage status, identify the chondral defect, and harvest chondrocytes from a non-weight-bearing zone of the distal femur [25,43,104,105]. Autologous chondrocytes are subsequently extracted, cultivated, and expanded in vitro, typically in an external laboratory. The expansion phase is usually performed in certified laboratories under standardised manufacturing conditions, and its duration may vary depending on the specific culture protocols. The time required also depends on the proliferation rate of the harvested chondrocytes and the total number of cells needed, which is influenced by the surface area and morphology of the defect. In a second-step surgery, the defect is debrided, and the membrane is secured into the defect. In the first-generation ACI procedures, a periosteal flap (pACI) was adopted to protect the chondrocytes from the joint cavity; however, pACI resulted in a high rate of graft hypertrophy [106]. In the second generation ACI, a II-III collagen membrane (cACI) was used to cover the defect [107]; however, this technique did not receive approval in some countries, and, therefore, did not achieve widespread adoption. Matrix-induced autologous chondrocyte implantation (mACI) represents the most widely adopted third-generation cell-based cartilage repair technique (Figure 3) [42,108,109,110,111,112,113,114,115,116,117]. In contrast to earlier iterations, mACI uses a biodegradable scaffold, typically composed of porcine-derived collagen I and III, into which autologous chondrocytes are seeded before in vitro expansion. This scaffold–chondrocyte construct is secured arthroscopically or via mini-arthrotomy in the defect, eliminating the need for periosteal flaps or sutures.

A growing body of evidence supports the use of mACI in medium-to-large defects, ranging from 2 to 10 cm^2^, particularly in young, active individuals with isolated unipolar lesions. A recent meta-analysis of 21 studies (1699 patients) reported significant improvements in IKDC, Lysholm, and KOOS scores over 2 to 5 years, with clinical benefit consistently exceeding the MCID [118]. Moreover, histological analysis and second-look arthroscopies have confirmed that MACI can achieve a high degree of tissue integration and partial restoration of hyaline-like properties, as indicated by MOCART scores, which show progressive maturation over time. Notably, the complication rate remains low, with graft hypertrophy and delamination observed in less than 10% of cases in modern series. Long-term (up to 10 years) durability has been demonstrated, particularly in lesions of the femoral condyles. A further development of mACI is spheroid-based systems such as Spherox, where chondrocytes are cultured in a three-dimensional matrix-free environment to form spheroids that secrete their extracellular matrix before implantation [100,119]. These are directly applied to the defect site and allowed to integrate within the cartilage bed [120,121,122,123,124,125]. Regulatory authorities, including NICE, have endorsed this technique for full-thickness cartilage defects of 2–10 cm^2^, particularly in the femoral and patellar regions. Early trials suggest comparable functional outcomes to scaffold-based mACI, with possible advantages in terms of cell viability, ease of implantation, and reduced operative time. However, long-term comparative evidence is still limited.

In parallel, one-step techniques have gained attention for their potential to reduce surgical burden while preserving biological potential. Among these, autologous minced cartilage implantation has emerged as a promising option, particularly for lesions measuring less than 3 cm^2^ [53,126]. In this approach, healthy cartilage harvested from low-load areas of the joint is mechanically minced into small fragments and implanted directly into the defect, often with the aid of a fibrin carrier and covered with a membrane [29,127]. This technique relies on the release of viable chondrocytes and chondrons from the matrix during fragmentation, stimulating endogenous repair while maintaining the zonal architecture of hyaline cartilage. Several recent studies have demonstrated functional improvements in KOOS, Tegner, and IKDC scores comparable to those of early mACI, with a favourable safety profile and the advantage of avoiding cell culture [50,128]. Nonetheless, concerns remain regarding the reproducibility of outcomes, graft stability, and integration in larger defects. Another frontier involves the use of mesenchymal stem cells (MSCs), which can be derived from BMAC, adipose tissue, or synovial fluid. These cells can be delivered via direct injection, incorporated into scaffolds, or combined with biological carriers such as hyaluronic acid or platelet-rich plasma [129,130]. The rationale lies in the multilineage differentiation potential and paracrine effects of MSCs, which may enhance chondrogenesis and modulate inflammation [131,132]. Although preclinical studies and early clinical trials yielded promising results, including improved histological quality and reduced pain, current evidence remains insufficient to establish MSC-based treatments as routine alternatives to established techniques. Moreover, heterogeneity in cell source, concentration, scaffold material, and outcome measures impedes direct comparison across studies.

Among the most debated indications for scaffold- and cell-based cartilage repair are those involving the patellofemoral joint. Lesions in the patella or trochlea are biomechanically and clinically distinct, given their exposure to higher shear forces, variable tracking, and coexisting instability. Historically, such defects have been considered less suitable for restorative techniques. However, recent studies suggest that, with appropriate patient selection and correction of concomitant malalignment or instability, favourable outcomes can be achieved. A previous systematic review evaluated mACI in the patellofemoral joint, demonstrating comparable improvements in KOOS and Tegner scores to those with femoral condyle defects, although with a slightly higher revision rate [36]. Similarly, the Spherox Phase III trial included patients with trochlear and patellar lesions, and demonstrated non-inferior outcomes compared to those with condylar lesions in terms of pain reduction and function. Nonetheless, residual maltracking and trochlear dysplasia were associated with poorer outcomes, underscoring the importance of a comprehensive biomechanical evaluation before surgical intervention.

Scaffold-assisted procedures are increasingly being explored in salvage scenarios, such as revision of failed microfracture, treatment of bipolar (kissing) lesions, and management of multifocal chondral pathology. While unipolar lesions remain the ideal target, bipolar and multipolar lesions have shown encouraging mid-term results when alignment, ligamentous stability, and meniscal integrity are restored in a staged or combined approach. In these complex cases, cell-based and scaffold-assisted techniques must be tailored not only to the lesion but to the overall joint environment, to optimise integration and avoid early failure [133].

## 6. Comparative Analysis of Cartilage Regeneration Techniques

The comparative evaluation between bone marrow-stimulating procedures and scaffold-based techniques has become a central topic in the evolving landscape of cartilage repair. Traditional bone marrow stimulation, particularly microfracture, has long been the first-line strategy for small focal defects given its simplicity, cost-effectiveness, and minimal invasiveness. However, concerns regarding the quality of the fibrocartilaginous repair tissue, progression to subchondral bone alterations, and deterioration of clinical outcomes over time have stimulated the development of more biologically robust alternatives. Among the enhanced BMS strategies, AMIC represents a significant step forward, as it combines microfracture with the implantation of a collagen membrane to stabilise the clot and favour mesenchymal stem cell retention and chondrogenic differentiation. Multiple clinical studies, including prospective trials and systematic reviews, have shown that AMIC provides superior mid- to long-term outcomes compared to microfracture alone, especially in borderline lesions (1–2 cm^2^), where traditional algorithms recommend microfracture; in these cases, AMIC has demonstrated more stable results, supporting its use as a reliable and cost-effective one-step procedure in focal chondral defects of the tibiofemoral and patellofemoral joints [11,46,47,48,134]. The same authors conducted two clinical trials comparing AMIC with microfractures for borderline-sized chondral defects in the tibiofemoral and patellofemoral joints. In both trials, the AMIC procedure achieves greater IKDC and Lysholm scores, as well as a significant reduction in the VAS score, in the management of patellar chondral defects. AMIC also resulted in a higher level of sports activity and a lower rate of failure at approximately four years of follow-up [47,48]. Similar results were reported in a recent meta-analysis of 18 comparative clinical studies (548 patients) with approximately four years of follow-up [11].

Scaffold-based and cell-based techniques such as mACI, spheroid technology, and gel-based ACI represent more complex and expensive approaches aimed at regenerating hyaline-like tissue. These strategies have shown excellent results in mid-size and large lesions, particularly in high-demand or previously treated patients. A critical analysis of registry data and comparative trials revealed that mACI leads to superior histological quality and mechanical resistance of the repair tissue, with better integration and durability. Scaffold-based ACI also appears to be less affected by subchondral bone remodelling, an issue frequently observed after microfracture or even AMIC in lesions exceeding 3 cm^2^. Emerging one-step options, including minced cartilage autografts, chondron-based injections, or stem cell-loaded matrices, have been proposed as simplified biological solutions that bridge the gap between bone marrow-stimulating procedures and ACI. However, the clinical evidence remains limited and heterogeneous, with short follow-ups, variability in carrier matrices, and often unclear indications. Comparative trials are scarce, and most data derive from single-centre series or early-phase studies. Unlike mACI, which uses laboratory-expanded autologous chondrocytes, AMIC is a single-session procedure that exploits the regenerative potential of bone marrow-derived mesenchymal stem cells. AMIC is more cost-effective, since it requires only one surgical step, avoiding in vitro cell expansion. Moreover, along with the avoidance of chondrocyte harvesting, AMIC should lead to less morbidity and faster recovery. These features make AMIC an attractive option for both surgeons and patients. A previous systematic review of 45 clinical studies (1667 procedures) compared to AMIC and mACI in the knee [42]. The AMIC group demonstrated greater values of IKDC and Lysholm scores and a lower rate of failures at approximately 40 months of follow-up [42]. A recent Bayesian network meta-analysis compared AMIC, ACI generations, and microfractures, as well as other techniques, for managing chondral defects of the knee [43]. Data from 2220 procedures (36 articles) were retrieved, with a median follow-up of three years [43]. AMIC resulted in higher Lysholm and Tegner scores, and the lowest rate of failures [43]. As expected, microfracture procedures were associated with a lower rate of hypertrophy [43]. A fundamental consideration in this comparative landscape is that technique selection should not rely solely on defect size. Morphology (kissing vs. unipolar), chronicity, location (patellofemoral vs. tibiofemoral), and patient factors (age, BMI, activity level) critically influence the choice of technique. A previous systematic review of 184 articles (8905 procedures) found that clinical outcomes were mainly related to the patients’ performance status before surgery [39]. A greater BMI was associated with a higher rate of hypertrophy [39]. Female sex and older age had a fair influence, while symptom duration before surgical intervention and cartilage defect size showed no association with the surgical outcome [39]. Lesion size and symptom duration did not show any association with the surgical outcome [39]. For instance, while AMIC is suitable for a broad range of focal lesions, mACI might be preferred in young active individuals with larger defects. In contrast, microfracture remains acceptable only for small, well-contained lesions in low-demand settings. Ultimately, the growing body of evidence supports a shift toward more biological and durable solutions such as AMIC, while underscoring the need for long-term data, comparative trials, and cost-effectiveness analyses to refine current algorithms.

## 7. Technical Considerations in Cartilage Repair

The method of membrane fixation in AMIC and scaffold-based techniques substantially influences construct stability and biological integration. Fibrin glue is the most widely used, given its simplicity and compatibility with arthroscopic techniques, but in vitro biomechanical tests have consistently shown lower fixation strength compared to sutures, especially under shear stress. Suture fixation, though more invasive, offers greater resistance to membrane displacement, particularly for uncontained or high-load lesions. However, suturing produces partial-thickness lesions of the articular cartilage [135]. These fissures may not heal and enlarge over time, potentially leading to surgical failure [136,137]. On the other hand, in translational pre-clinical studies, fibrin glue impairs the migration and proliferation of mesenchymal stem cells and chondrocytes in porcine-derived collagen I/III membranes, commonly employed in mACI and AMIC [37,41,138]. A recently published study compared 26 studies (1539 procedures) reporting outcomes on suture, fibrin glue, and no fixation among studies on ACI [135]. The authors found that no membrane fixation resulted in a statistically significantly lower rate of revision surgeries and a higher IKDC score [135]. Additionally, avoiding fixation reduces costs and surgical time. Finally, fibrin degrades within weeks, and may be insufficient to resist early mobilisation. Taken together, this evidence supports that the membrane should not be fixed into the defect. Clinical studies are needed to validate this evidence in a clinical setting.

Access to the defect has evolved from open techniques toward increasingly less invasive approaches. While classical ACI and early AMIC procedures often required full arthrotomy, especially for patellofemoral or multifocal lesions, current trends favour mini-arthrotomy or fully arthroscopic techniques. Arthroscopy reduces soft tissue trauma, facilitates faster rehabilitation, and is compatible with AMIC and mACI spheroids. While there is general agreement that arthrotomy is excessively invasive, the debate remains open regarding the use of mini-arthrotomy and arthroscopy. A recent systematic review compared 16 studies (770 procedures) reporting outcomes on arthroscopy and mini-arthrotomy [14]. No differences were found in terms of Tegner score, Lysholm score, IKDC, and rates of failures and revisions. These results indicated that both arthroscopy and mini-arthrotomy approaches for mACI in the knee achieve similar outcomes at a four-year follow-up. However, for patellar defects or large uncontained areas, arthrotomy or mini-arthrotomy may still be necessary to allow accurate membrane placement and fixation.

The Magnetic Resonance Observation of Cartilage Repair Tissue (MOCART) score is widely used for semi-quantitative MRI-based evaluation of cartilage repair. It assesses parameters such as defect fill, surface integrity, integration with adjacent cartilage, and signal intensity. While MOCART provides structured imaging endpoints, its correlation with clinical outcomes remains debated. Recently, investigations on AMIC and mACI have highlighted cases with excellent MOCART values. Still, only modest improvements in PROMs, and vice versa, underscore that imaging appearance does not always reflect biomechanical or symptomatic recovery. Furthermore, MOCART lacks sensitivity to tissue quality at the microscopic level and fails to capture the functional properties of cartilage. Following this observation, a recent multivariate analysis of 1017 procedures revealed that the MOCART score demonstrated no association with patient characteristics or surgical outcome in patients who underwent surgical management for knee and talus chondral defects [13]. Despite these limitations, MOCART remains useful to monitor graft maturation, particularly in comparative trials. Newer 3D compositional MRI techniques and quantitative T2/T1ρ mapping may enhance future evaluations, but their adoption in clinical practice remains limited.

## 8. Biomechanical and Surgical Considerations in Patellofemoral and Tibiofemoral Lesions

The patellofemoral and tibiofemoral compartments exhibit distinct anatomical, biomechanical, and functional properties, which critically influence both lesion pathophysiology and the outcomes of cartilage repair. As such, strategies effective in one compartment cannot be indiscriminately applied to the other without considering their unique loading conditions, joint kinematics, and surgical access requirements. From a biomechanical perspective, the patellofemoral joint sustains high compressive forces during activities that involve knee flexion beyond 60°, particularly during stair ascent, squatting, or rising from a seated position. Peak contact pressures in the patellofemoral joint may exceed 6 MPa, often localised in narrow contact zones. Additionally, the articular surface of the patella is subjected to tangential shear during tracking, especially in malaligned or dysplastic knees. These conditions produce a mechanically hostile environment for scaffold integration and tissue maturation, particularly in central lesions. In contrast, the tibiofemoral joint exhibits broader contact areas and more stable loading vectors, with lower peak stresses under physiological conditions. However, malalignment, ligament deficiency, or meniscal compromise may dramatically alter this equilibrium. Histologically, patellofemoral cartilage has a thinner superficial zone and a less organised collagen fibre orientation than tibiofemoral cartilage, making it less resistant to repeated shear and compression. Furthermore, patellofemoral lesions are more frequently associated with instability, dysplasia, or increased lateral tilt, conditions that may impair the mechanical protection of the repair site and increase the risk of failure. Clinical outcomes after cartilage repair confirm this difference: multiple studies report lower PROM improvements and higher revision rates following mACI or AMIC in the patellofemoral compartment compared to isolated condylar lesions, unless concomitant realignment or patellofemoral optimisation procedures are performed [48,90,138,139,140,141,142,143,144,145]. Surgically, access to the patellofemoral joint presents challenges. Central or lateral patellar lesions are difficult to visualise and prepare arthroscopically, often requiring arthrotomy. Miniarthrotomy with patellar eversion remains the standard to achieve perpendicular access and precise membrane fixation. This contrasts with tibiofemoral lesions, which are increasingly treated via all-arthroscopic or mini-open approaches using cannulated or injectable systems. In vitro models have demonstrated that perpendicular scaffold placement in the patellofemoral compartment is critical for mechanical stability, as tangential application leads to early delamination and edge lifting under simulated motion. Fixation techniques also differ. While glue alone may suffice in the femoral condyles, patellofemoral lesions, particularly on the patellar ridge or lateral facet, often benefit from augmentation to resist multidirectional shear.

## 9. Gel-Based Constructs, Simplified Workflows, and Biological Innovation

Future research should focus on high-quality prospective registries, ideally multicentric and stratified by lesion morphology, anatomical site, and surgical technique. Head-to-head comparisons using standardised PROMs and failure definitions are essential to refine indications and predict long-term durability. Special attention should be given to the patellofemoral compartment, kissing lesions, and multipolar defects, which remain under-represented yet highly relevant in clinical practice, with challenging management and unpredictable results. Ultimately, a shift toward patient-specific, morphology-driven algorithms is needed to replace the current one-size-fits-all approach, integrating both clinical and biomechanical considerations into surgical planning. In response to the increasing demand for efficient, biologically effective and technically reproducible cartilage repair solutions, the field is rapidly evolving toward next-generation techniques that aim to simplify surgical workflows without compromising tissue quality. Among these, injectable or mouldable gel-based constructs have garnered considerable interest. These systems, often composed of hydrogel matrices functionalised with growth factors, autologous chondrocytes, mesenchymal stem cells, or chondrons, offer a one-step arthroscopic delivery method with potential for homogeneous defect coverage and enhanced cell viability. From a biomechanical standpoint, gel-based matrices must reconcile conflicting requirements: they must be soft enough to conform to complex defect geometries and permit nutrient diffusion yet resilient enough to resist early shear forces and joint compression. Preliminary rheological and indentation studies suggest that current hydrogels still fall short of collagen membranes in terms of stiffness and tensile strength; however, newer crosslinking strategies and hybrid scaffolds have shown improvements in mechanical performance [146,147,148,149]. Biomaterial viscoelasticity exerts a significant influence on integration and load transmission, particularly in high-demand compartments, such as the patellofemoral joint. Biologically, gel-based approaches may improve the local microenvironment by promoting a more uniform cell distribution and controlled release of trophic factors. Experimental models have demonstrated that such matrices facilitate chondrogenic differentiation and extracellular matrix deposition, often surpassing traditional techniques in terms of type II collagen content and integration at the cartilage–bone interface. However, long-term human data remain scarce, and many products are still in preclinical or early-phase trials.

Another promising direction involves the use of chondron aggregates and microfragmented cartilage tissue, which preserve native pericellular matrix components and chondrocyte niches. These have shown encouraging results in terms of histological integration and symptom relief, particularly when used in minimally manipulated, one-step settings. Bioprinting and gene-editing technologies, though still experimental, may further personalise constructs, matching patient-specific defect geometry and biological requirements. Overall, while scaffold-based and cell-laden procedures remain the gold standard in selected indications, the future is moving toward biologically active, technically streamlined and cost-conscious approaches. Nevertheless, rigorous biomechanical validation and prospective clinical trials will be essential to translate these innovations into robust, reproducible outcomes.

## 10. Limitations

Despite substantial advancements in the surgical management of focal chondral defects, several limitations persist within the current body of evidence, affecting both the reliability of clinical recommendations and the generalisability of reported outcomes. The majority of available studies consist of case series or cohort analyses, often monocentric and non-randomised, which inherently limit the level of evidence and introduce potential biases in patient selection, follow-up adherence, and the reporting of complications. Randomised controlled trials comparing different surgical techniques are scarce, and even fewer studies provide long-term follow-up beyond 5 to 10 years. This gap becomes particularly relevant considering the delayed failure patterns often observed in biological procedures, especially in active populations. Outcome measures remain a source of significant heterogeneity. Although PROMs, such as KOOS, IKDC, Lysholm, and Tegner scores, are widely used, they vary in terms of sensitivity, responsiveness, and relevance depending on the patient’s profile and lesion characteristics. Furthermore, many studies aggregate data across heterogeneous anatomical sites, including condylar, trochlear, and patellar defects, without adequately stratifying results by location, despite the known biomechanical and biological differences among compartments. The same concern applies to lesion morphology, as kissing lesions and multifocal defects are frequently excluded or under-reported, leading to a systematic bias in the interpretation of clinical efficacy. These lesion types represent particularly challenging scenarios, characterised by lower biological potential and altered joint mechanics, yet the current literature provides limited guidance regarding their optimal management. Few studies address whether treating one or both surfaces yields superior outcomes, and the role of alignment correction or meniscal preservation is often insufficiently controlled. Another critical limitation involves the lack of standardisation in surgical indications, perioperative protocols, and rehabilitation strategies. Studies usually apply arbitrary size cut-offs, typically around 2 or 2.5 cm^2^, to guide the selection of techniques such as microfracture, AMIC, or MACI. However, several recent reports have demonstrated favourable outcomes with scaffold-augmented techniques even in borderline or small defects, challenging traditional algorithms. Moreover, the impact of patient-specific factors, such as age, BMI, activity level, or chronicity, is inconsistently addressed or reported, which impairs the development of morphology-informed treatment paradigms. Emerging technologies such as one-step cell therapies, stem cell-loaded scaffolds, and biological adjuvants show promise but suffer from methodological inconsistencies and regulatory constraints that hinder their widespread adoption. Comparative effectiveness studies are rare, and cost-effectiveness analyses remain largely speculative, limiting evidence-based policymaking.

## Figures and Tables

**Table 1:** Strings used for the search in each database (WoS: Web of Science).

Database	Search String
PubMed	(“knee” [MeSH Terms] OR “knee joint” [MeSH Terms]) AND (“cartilage, articular” [MeSH Terms] OR “cartilage repair” OR “chondral defect” OR “focal cartilage lesion”) AND (“autologous chondrocyte implantation” [MeSH Terms] OR “ACI” OR “AMIC” OR “bone marrow stimulation” OR “scaffold” OR “cell therapy” OR “regenerative medicine”)
Scopus	TITLE-ABS-KEY(knee AND (cartilage OR chondral) AND (ACI OR AMIC OR “bone marrow stimulation” OR scaffold OR “cell therapy” OR “regenerative surgery”))
WoS	TS = (knee AND (cartilage OR chondral OR “focal defect”) AND (ACI OR AMIC OR “bone marrow stimulation” OR scaffold OR “cell-based therapy” OR “regenerative approach”))

## Data Availability

The datasets generated during and/or analysed during the current study are available throughout the manuscript.

## Abbreviations

The following abbreviations are used in this manuscript:

ACI	Autologous Chondrocyte Implantation
AMIC	Autologous Matrix-Induced Chondrogenesis
BMAC	Bone Marrow Aspirate Concentrate
BMI	Body Mass Index
cACI	Collagen-covered Autologous Chondrocyte Implantation
IKDC	International Knee Documentation Committee
IL-1β	Interleukin-1 beta
KOOS	Knee Injury and Osteoarthritis Outcome Score
mACI	Matrix-induced Autologous Chondrocyte Implantation
MCID	Minimum Clinically Important Difference
MMPs	Matrix-degrading Enzymes
MOCART	Magnetic Resonance Observation of Cartilage Repair Tissue
MRI	Magnetic Resonance Imaging
MSCs	Mesenchymal Stem Cells
NICE	National Institute for Health and Care Excellence
pACI	Periosteal flap Autologous Chondrocyte Implantation
PROMs	Patient-Reported Outcome Measures
TNF-α	Tumour Necrosis Factor-alpha
VAS	Visual Analogue Scale
